# The Study of Expression of Hypoxia-Inducible Factor-1 Alpha (HIF-1 Alpha) and Hypoxia-Inducible Factor-2 Alpha (HIF-2 Alpha) in Oral Squamous Cell Carcinoma: An Immunohistochemical Study

**DOI:** 10.7759/cureus.45189

**Published:** 2023-09-13

**Authors:** Niva Mahapatra, Abikshyeet Panda, Kailash Dash, Lipsa Bhuyan, Pallavi Mishra, Aishwariya Mohanty

**Affiliations:** 1 Oral and Maxillofacial Pathology, Kalinga Institute of Dental Sciences, Bhubaneswar, IND; 2 Oral Pathology, Kalinga Institute of Dental Sciences, Bhubaneswar, IND; 3 Oral Pathology, Srirama Chandra Bhanja (SCB) Dental College and Hospital, Cuttack, IND

**Keywords:** immunohistochemistry, hypoxia inducible factor, angiogenesis, tumorigenesis, oral squamous cell carcinoma

## Abstract

Context: Oral cancer is the major cause of mortality and morbidity worldwide. There are many factors that influence the tumor microenvironment that promotes tumorigenesis. Hypoxia is one of the factors that affects the process of angiogenesis by inducing proangiogenic factors to maintain the blood supply which in turn enhances the aggressiveness of the tumor and prognosis of solid tumors such as oral squamous cell carcinoma.

Aim and objective: The aim of the study was to compare the expression of hypoxia-inducible factor 1α (HIF-1α) and hypoxia-inducible factor 2α (HIF-2α) in various histological grades of oral squamous cell carcinoma immunohistochemically.

Methodology: Immunohistochemical evaluation of HIF-1α and HIF-2α was done in 90 samples of oral squamous cell carcinoma which were graded histologically into 30 samples each of well, moderately and poorly differentiated squamous cell carcinoma.

Statistical evaluation: Statistical analysis was done to study the prognostic significance of the biomarkers.

Results: All the cases showed positivity for expression of HIF-1α and HIF-2α. The number of positive staining in both markers reduced as the tumor severity increased from well to poorly differentiated. The expression of MIL of HIF-2α was higher than HIF 1α and HIF 2α expression was mostly seen in cytoplasmic in well-differentiated and nuclear in both moderately and poorly differentiated OSCC suggestive that HIF-2α is a more specific marker to hypoxia.

Conclusion: Hypoxia is an essential factor that triggers other angiogenic switch and inflammatory factors which facilitates the process of tumorigenesis. This is also important for predicting the treatment outcome and prognosis of the patients. HIF-2α is a more sensitive marker that appears to be correlated and could perhaps serve as a good surrogate marker of hypoxia.

## Introduction

Cancer of the head and neck region ranks as the seventh most common neoplasm worldwide. Among these 90% of cases accounts for oral squamous cell carcinoma (OSCC) [1,2]. The presence of premalignant lesions like oral squamous dysplasia, of variable severity typically graded on a 3-point scale (grade 1 to 3) predisposes to transformation into malignancy and the rate of conversion varies from 3% to 33%. The hallmarks of oral cancer suggest that oral cancer is multifactorial and alters the tumor microenvironment (TME) [3]. This modified TME results in the upregulation and production of different molecules that enhance the tumor progression. In solid tumors like OSCC, the malignant cells proliferate exponentially which causes an imbalance in the oxygen supply due to poor vascular architecture of the neo-angiogenic vessels. This insufficient oxygen concentration (pO_2_ < 10 mmHg) in neoplastic cells results in hypoxia as it exceeds the required angiogenic demand [4,5]. Hypoxia is the most common characteristic of solid tumors enabling them to adapt and survive in TME with reduced oxygen concentration as severe hypoxia causes cell death in normal conditions [6].

Tumor hypoxia is meditated by hypoxia-inducible factors (HIF 1, HIF 2, and HIF 3) that regulate the process of tumorigenesis, progression, and prognosis to cancer therapy. The HIFs facilitate the malignant cells to adapt in oxygen-deprived environment by altering the cellular metabolism, evading apoptosis, and regulating the expression of genes that promote the proliferation of neoplastic cells. The HIF 1 alpha and 2 alpha roles are very distinctive but HIF-1α majorly mediates the transcription of many genes in cancer [7]. HIF 1 is composed of two subunits namely HIF-1α and HIF-1β. HIF-1β is a constitutively nuclear subunit that is expressed under all oxygen conditions. HIF-1α is a highly regulated subunit that contains a unique oxygen-dependent degradation domain (ODDD) that maintains the stability of this protein centrally. Under normoxia, this protein is highly labile with a half-life of less than 5 minutes. In oxygen deprivation the degradation of HIF-1α by proteasome is blocked that stabilizes this protein and further causes its dimerization with HIF-1β to form an active HIF-1 complex. HIF-1α is implicated in mutation in tumor suppressor genes like VHL, p53 and expression of oncogenes like Akt and Ras alter the angiogenic switch (enhances VEGF expression) resulting in neovascularization in low oxygen concentration that promotes growth, metastasis, and invasion of tumoral cells resulting in poor outcome in cancer therapy and increased mortality [6,8-11]. HIF-2α named as (EPAS 1/HRF/HLF/MOP2) is a mammalian basic helix‑loop‑helix per‑aryl hydrocarbon receptor nuclear translocator (ARNT) ‑ Sim (bHLH‑PAS) protein similar to HIF‑1α. Both HIF‑1α and HIF‑2α overlap structurally and have 48% similarity in amino acid identity [12]. HIF‑2α binds to ARNT and transactivates the hypoxia‑responsive genes that target erythropoietin (EPO) and VEGF that promote tumorigenesis and neovascularization and progression in solid tumors [13]. Expression of HIF-2α is very specific to certain cell types viz fibroblasts of the kidney, type II pneumocytes, endothelial cells, interstitial cells of the duodenum, and pancreas [7].

In this context, the present study aimed to study the expression of hypoxia-inducible 1α (HIF-1 alpha) and hypoxia-inducible 2α (HIF-2 alpha) antibodies in various histological grades of OSCC immunohistochemically to understand its prognostic significance.

## Materials and methods

Patients and tissue samples

In this retrospective study, 90 formalin fixed paraffin embedded archival tissue blocks were obtained from the Department of Oral Pathology and Microbiology, Kalinga Institute of Dental Sciences and Hospital, Bhubaneswar. The samples were divided into three groups as under:

Group I: (30 samples) - Well-differentiated OSCC

Group II: (30 samples) - Moderately differentiated OSCC

Group III: (30 samples) - Poorly differentiated OSCC

The approval for the study was obtained from the Institutional Ethics Committee of Kalinga Institute of Dental Sciences, Bhubaneswar with letter number KIIT/KIMS/IEC/554/2021.

Demographic details of all patients like age, gender, and history of the presence of deleterious habits such as alcohol consumption and tobacco (chewing/smoking) were recorded.

Immunohistochemistry

**Figure 1:**
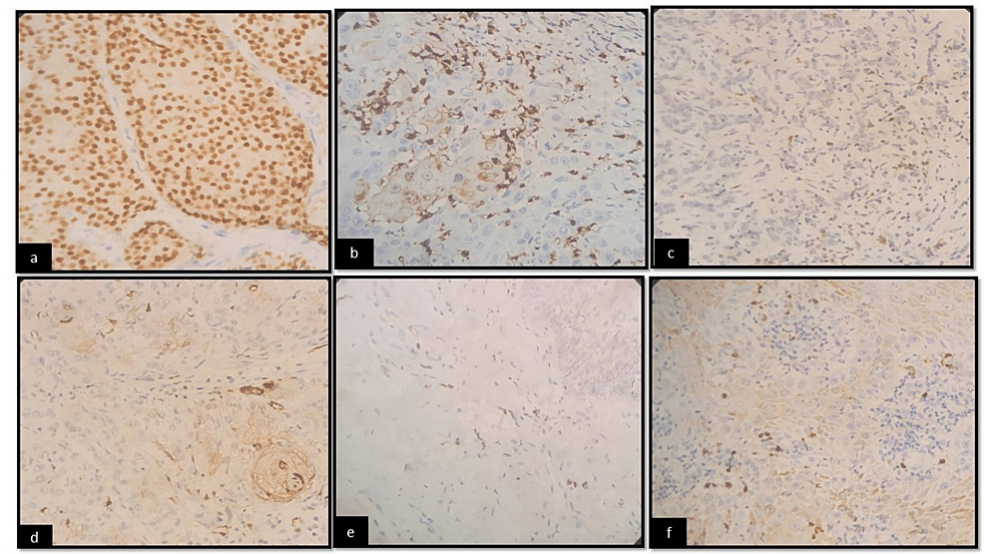
The immunohistological staining shows (a) HIF-1α immunostaining in well-differentiated squamous cell carcinoma (*100), (b) HIF-1α immunostaining in moderately differentiated squamous cell carcinoma (*100), (c) HIF-1α immunostaining in poorly differentiated squamous cell carcinoma (*100), (d) HIF-2α immunostaining in well-differentiated squamous cell carcinoma (*100), (e) HIF-2α immunostaining in moderately differentiated squamous cell carcinoma (*100), (f) HIF-2α immunostaining in poorly differentiated squamous cell carcinoma (*100). Courtesy: Department of Oral and Maxillofacial Pathology, Kalinga Institute of Dental Sciences, Bhubaneswar HIF: hypoxia-inducible factor

Three micron-thick serial sections were cut from formalin‑fixed paraffin‑embedded blocks and transferred onto Poly-L-Lysine coated positively charged slides. Antigen retrieval was done by transferring the slides to Tris EDTA buffer of pH 9 in the pressure cooker at 105 degrees Celsius for 20 min. The slides were treated with 3% hydrogen peroxidase for 10 min to quench endogenous peroxidase activity. Prediluted primary antibodies, namely rabbit polyclonal HIF-1α antibody (EP118, biogenix) and HIF-2α antibody (ARB28-5R, biogenix) were added to the sections and incubated for 60 min in a hydrated chamber. The sections were then washed with immunowash buffer (prepared by diluting 1 part of 25X immunowash buffer in 24 parts of distilled water) for 2-3 min and a super‑enhancer reagent was added and incubated for 20 min. Then, the sections were washed with immune wash buffer for 2-3 min and incubated for 30 min with a secondary antigen that is polymer-horseradish peroxidase immunohistochemistry, 3,3′ (HRP IHC) detection system. Chromogen diaminobenzidine (DAB) was added and incubated for 10 min, then washed with distilled water for 3 min and counterstained with hematoxylin. Negative control sections were omitted from the primary antibody. Breast cancer specimens known to express HIF-1 alpha-positive cells served as the control group and lung cancer specimens known to express HIF-2 alpha-positive cells served as the control group. HIF-1α and HIF-2α staining were seen in the cytoplasm and nucleus in hypoxic malignant cells (Figures 1a-1f).

Evaluation of slides

Quantitative Analysis

The immunostained slides of various histopathological grades (well, moderate, and poorly differentiated) of OSCC were evaluated for HIF-1α and HIF-2α antibodies. Cells were considered immunopositive if they presented brown cytoplasmic staining regardless of intensity and were examined under high power (400x) magnification. HIF-1 alpha and HIF-2 alpha positive cells were counted 1000 cells/entire section randomly selected microscopic fields.

The mean labeling index (MLI) for all the positive groups was calculated using the formula [2]:

(Number of positive cells/Total number of cells) X 100

Qualitative Analysis

The immunostained slides were then graded into three categories based on the intensity of the staining. Each case was graded as (+) mild, (++) moderate, and (+++) intense stain by two blinded observers independently with respect to the positive control.

Statistical analysis

The entire data was entered using MS Excel 2016 (Microsoft, USA) and analyzed using Statistical Package for the Social Sciences (IBM SPSS Statistics for Windows, IBM Corp., Version 25.0, Armonk, NY). Descriptive statistics were calculated for the continuous variable and represented using mean and standard deviation. Continuous variables were represented using frequencies and percentages and represented in graphical patterns. One was the analysis of variance (ANOVA) statistics used to calculate the inferential statistics. Reliability statistics in terms of Kappa value were done using cross tabs to study the prognostic significance of the biomarkers. Statistical significance was fixed at 0.05; any value below 0.05 was considered statistically significant.

## Results

**Figure 2:**
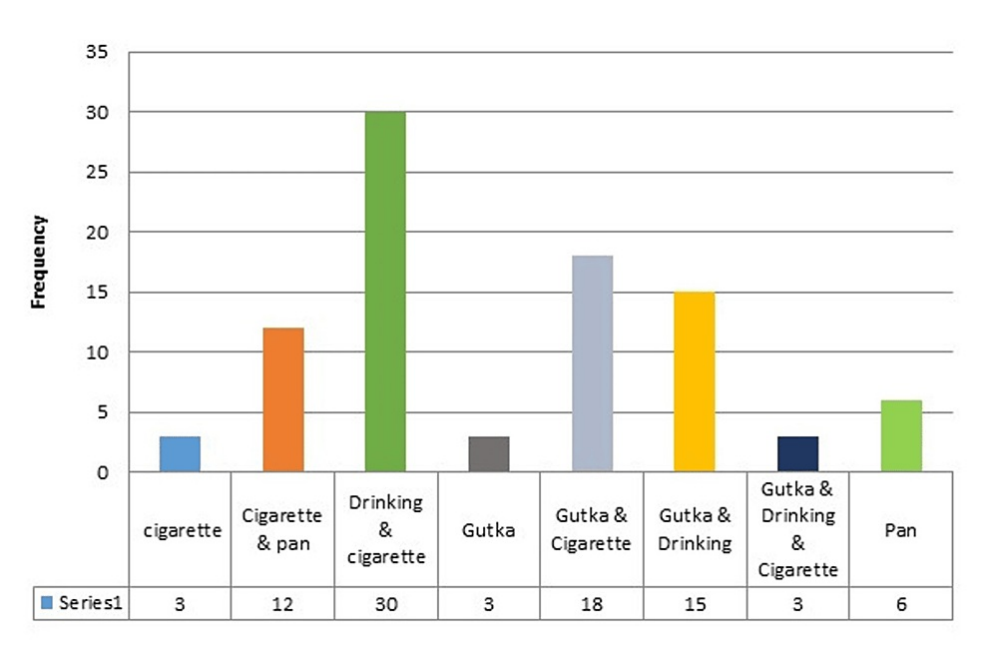
Distribution of the habit for the study population

The mean age of the study population was 59.17±7.772. There were 81 (90%) males and nine (10%) females. The distribution of the habits was also graphically represented in Figure 2. There was a statistically significant difference observed for in-category variations for the gender group and habits (p<0.001). The highest prevalence of drinking along with cigarette smoking habits was observed.

Evaluation of mean labeling index (MLI) of HIF-1α and HIF-2α

The presentation of the data was tabulated for comparing the MLI between HIF-1 alpha and HIF-2 alpha in different study groups of OSCC. The mean score for HIF-2 was higher for well and poorly differentiated OSCC, while HIF-1 was noted to be higher for moderately differentiated OSCC. A statistically significant difference was noted (p<0.001) (Table 1).

**Table 1:** Comparison of a mean labeling index between HIF-1 alpha and HIF-2 alpha in different study groups of OSCC OSCC: oral squamous cell carcinoma, HIF: hypoxia-inducible factor

Categorization	Group	N	Mean	Std. Deviation	95% Confidence Interval for Mean		F Score	P Value
					Lower Bound	Upper Bound		
Well-differentiated carcinoma	HIF-1	30	33.70	3.668	32.33	35.07	26.813	<0.001*
	HIF-2	30	38.00	2.982	36.88	39.11		
	Total	60	35.85	3.960	34.82	36.87		
Moderately differentiated carcinoma	HIF-1	30	26.25	0.858	25.92	26.57	48.207	<0.001*
	HIF-2	30	23.85	1.732	23.20	24.49		
	Total	60	25.05	1.817	24.58	25.51		
Poorly differentiated carcinoma	HIF-1	30	19.95	1.949	19.22	20.67	51.506	<0.001*
	HIF-2	30	23.10	1.487	22.54	23.65		
	Total	60	21.52	2.340	20.92	22.13		

The tissue localization for both the HIF-1 and HIF-2 has been mentioned. For HIF-1, there was a statistically significant difference noted for well (p=0.027) and moderately differentiated (p=0.028) lesions. HIF-1 marker showed that in well-differentiated carcinoma, the frequency of cytoplasmic representation was 4, cytoplasmic and nuclear was 16, and nuclear was 10. For moderately differentiated carcinoma, the frequency of cytoplasmic representation was 21, and nuclear was 9. A statistically significant difference was noted among the different tissue localization for both well and moderately differentiated lesion categories. For poorly differentiated lesions, the frequency of cytoplasmic representation was 14, cytoplasmic and nuclear was 3, and was highly distributed in the nuclear area that was 14. This finding is crucial as HIF-1 alpha is a transcriptional factor. There was no cytoplasmic and nuclear presentation for both the moderately and poorly differentiated categories. No statistically significant difference was noted between the distributions in the poorly differentiated carcinoma category (Table 2).

**Table 2:** Categorization and presentation of HIF-1α according to the tissue localization for individual groups HIF: hypoxia-inducible factor

Categorization		Frequency	Percent	F Score	P Value
Well-differentiated carcinoma	Cytoplasmic	4	13.3	7.200	0.027*
	Cytoplasmic+Nuclear	16	53.3		
	Nuclear	10	33.3		
	Total	30	100.0		
Moderately differentiated carcinoma	Cytoplasmic	21	70.0	4.800	0.028*
	Cytoplasmic+Nuclear	0	0		
	Nuclear	9	30.0		
	Total	30	100.0		
Poorly differentiated carcinoma	Cytoplasmic	14	46.7	0.133	0.715
	Cytoplasmic+Nuclear	0	0		
	Nuclear	16	53.3		
	Total	30	100.0		

HIF-2 marker showed that in well-differentiated carcinoma, the frequency of cytoplasmic representation was 10, cytoplasmic and nuclear was 6, and nuclear was 14. For moderately differentiated carcinoma, the frequency of cytoplasmic representation was 16, cytoplasmic and nuclear was 5, and nuclear was 9. For poorly differentiated lesions, the frequency of cytoplasmic representation was 12, cytoplasmic and nuclear was 3, and nuclear was 15. No statistically significant difference was noted between the distributions in each lesion category (Table 3).

**Table 3:** Categorization and presentation of HIF-2α according to the tissue localization for individual groups HIF: hypoxia-inducible factor

Categorization		Frequency (N)	Percent (%)	F Score	P Value
Well-differentiated carcinoma	Cytoplasmic	10	33.3	3.200	0.202
	Cytoplasmic+Nuclear	6	20.0		
	Nuclear	14	46.7		
	Total	30	100.0		
Moderately differentiated carcinoma	Cytoplasmic	16	53.33	2.400	0. 173
	Cytoplasmic+Nuclear	5	16.67		
	Nuclear	9	30.00		
	Total	30	100.0		
Poorly differentiated carcinoma	Cytoplasmic	12	63.33	1.600	0.212
	Cytoplasmic+Nuclear	3	10.00		
	Nuclear	15	26.67		
	Total	30	100.0		

The intra-observer reliability (Kappa) statistics with cross-tabular representation and McNemar statistics were done. It was observed that for HIF-1 alpha, the Kappa score was 1 denoting complete agreement and for HIF-2 alpha, the Kappa value was 0.822 which showed excellent agreement.

## Discussion

HIF is a hetero-dimeric protein made up of alpha (a) and beta (b) subunits. It promotes the transcription of genes related to angiogenesis, glucose/energy metabolism, cellular growth, metastasis, and apoptosis, giving solid tumors a survival adaptive advantage under hypoxic conditions. Many single nucleotide polymorphisms can influence the stability and susceptibility to inhibition of the HIF-1 alpha gene.

Oral malignancies develop throughout time as a result of gradual, sustained accumulations of mutations that change the normal mucosa from dysplasia to invasive carcinoma. One of the keys to lowering the mortality, morbidity, and treatment costs related to OSCC may lie in the identification of high-risk oral premalignant diseases and early intervention. The two that are most frequently seen are erythroplakia and leukoplakia. Solid malignant tumors, like OSCC, have the capacity for unrestricted and rapid growth. Hypoxia is a frequent symptom that leads to the spread of localized and systemic cancer, resistance to treatment, and unfavorable prognosis.

The control of transcription by HIF-1 is a key mechanism mediating adaptive responses to reduced oxygen availability (hypoxia), which occurs in many human malignancies relative to normal tissue (HIF-1). Neovascularization and enhanced glycolysis are hypoxic microenvironment-related changes that are linked to tumor invasion and metastasis. Unbalanced oxygen delivery and consumption leads to the development of a hypoxic microenvironment. Squamous cell carcinoma of the head and neck, which is quickly growing, has limited blood supply and insufficient vascularization. A variety of oncogenes, including HIF and vascular endothelial growth factor (VEGF), are upregulated in solid tumors in response to hypoxic stress, which promotes irregular vascular endothelial cell proliferation and differentiation. Many tumor forms have been shown to produce HIF-1, and this expression has been inconsistently linked to prognosis. The accuracy of HIF in the oral cavity's prognostic and diagnostic methods is poorly understood. In order to comprehend prognostic efficacy in various OSCC grades, this study was conducted.

The present immunohistochemical study was done using two markers of hypoxia belonging to hypoxia-inducible transcriptional factors namely HIF-1α and HIF-2α in three grades of OSCC (well, moderately, and poorly differentiated). HIFs are a basic loop helix loop protein that forms a heterodimeric complex resulting in the transcription of various genes that cause major metabolic changes under the oxygen-deprived TME. Both these markers are isoforms and share constitutively 48% of amino acid sequences. However, these markers have distinct expressions due to many internal and external factors that can induce transcription of overlapping or distinctive target genes [8]. Although elaborate studies have been done to study the expression of HIF-1α in solid neoplasms like OSCC in contrast to limited studies on its other isoform HIF-2α. Hence, these two markers were considered from various other endogenous markers of hypoxia.

The results demonstrated a considerable decrease in HIF-1α and HIF-2α protein expression in OSCC samples belonging to different severities. This is in contrast to other studies done by many authors [6-11,14,15]. The most probable explanation is the limited sample size in our study and more studies are required in cases of poorly differentiated carcinomas to understand the prognostic importance of HIF-1 alpha. The expression of the MLI of HIF-2α was higher than HIF-1α suggesting that HIF-2α is a more specific marker to hypoxia as suggested by authors. The divergence in the expression of HIF-1α and HIF-2α is mostly due to different pathways that result in the activation of the isoforms under hypoxic conditions [15,16]. The next probable explanation as stated by the authors is that HIF-2 alpha showed a more sustainable expression in hypoxia in solid tumors than HIF-1 alpha. Further, HAF, the receptor of activated protein kinase C (RACK1), causes oxygen-independent degradation of HIF-1 alpha and an increase in natural antisense HIF-1 alpha that prevents its transcription [17].

In our study, we found tissue localization of HIF-1α is mostly in cytoplasmic + nuclear in well-differentiated carcinoma and cytoplasmic in moderately differentiated carcinoma which was statistically significant, and HIF-2α expression was mostly seen in cytoplasmic in well-differentiated and nuclear in both moderately and poorly differentiated OSCC [10,13]. It was seen that the stains were positively visible for the biomarkers in both the cytoplasm and nuclear membrane majorly. Individual presentation of the genes was also noted. The novelty of the present study revolved around the fact that the present study highlighted the presentation of the stains and biomarkers for individual grades of the lesions and reported the Cohen’s Kappa statistics for comparing and studying the reliability of adapting this procedure as a prognostic method. This can be used as an adjunct marker for various grades of the neoplasm. The nucleo-cyoplasmic shuttling results in the binding of isoforms of HIF-1 alpha and HIF-2 alpha with importin alpha1, alpha3, alpha5, and alpha7. This is facilitated by nuclear transporter receptors that cause stabilization of HIF in the nucleus activating various target genes like VEGF, EPO, and glucose transporter 1 (GLUT1) [18]. Authors have also stated that inflammation also induces the TME of solid tumors [19]. The stabilization of HIF-1 alpha causes the recruitment of chemokines and its receptors that cause accumulation of tumor-activated macrophages (TAMS) that in turn promote tumor-promoting chronic inflammation hampering the adaptive immunity and activating factors that induce endothelial (increased expression of VEGF), malignant proliferation, and survival of malignant cells. HIF-2 alpha is also expressed on TAMS promoting infiltration and migration by the chemotactic mechanism of macrophages [20]. Hence, more studies are necessary to find the correlation between hypoxia and inflammatory mediators.

Any research has certain limitations. Our study also had certain limitations such as instead of using a single biomarker, a panel of endogenous hypoxia markers should be applied to correctly quantify the degree of hypoxia levels within a solid tumor such as OSCC. GLUT1, carbonic anhydrase IX, and VEGF should also be included in this panel of hypoxia markers in addition to HIF-1α and HIF-2α [16]. In order to evaluate the prognostic and diagnostic importance of HIF-2α overexpression and its co-expression in our population, we propose more extensive investigations.

## Conclusions

Head and neck squamous cell carcinoma (HNSCC) frequently exhibits hypoxia, which promotes the growth of tumorous aggression and metastasis. Treatment of HNSCC should focus heavily on the hypoxic situation. The poor prognosis of HNSCC has been overcome by increasing oxygen delivery, eliminating the hypoxic microenvironment, and increasing the pace at which hypoxic cells die by utilizing hypoxic cell radiosensitizer and hypoxic cell cytotoxic. In order to anticipate OSCC's prognosis and to help develop a customized treatment plan, HIF-2α expression in OSCC is therefore likely to be very valuable and is an adjunctive prognostic marker.
